# Supplementary material for: Single-shot 20-fold expansion microscopy
Source: Nat Methods. 2024 Oct 11;21(11):2128–34. doi: 10.1038/s41592-024-02454-9 (PMC11541206; doi:10.1038/s41592-024-02454-9)
Supplement: Supplementary file 1 — Supplementary Figs. 1–5, Tables 1–3 and Notes 1–9. [file 41592_2024_2454_MOESM1_ESM.pdf]

nature methods

Supplementary information https://doi.org/10.1038/s41592-024-02454-9

Single-shot 20-fold expansion microscopy

In the format provided by the authors and unedited

Table of Contents

Supplementary Figures 1-5
Supplementary Tables 1-3
Supplementary Notes 1-9

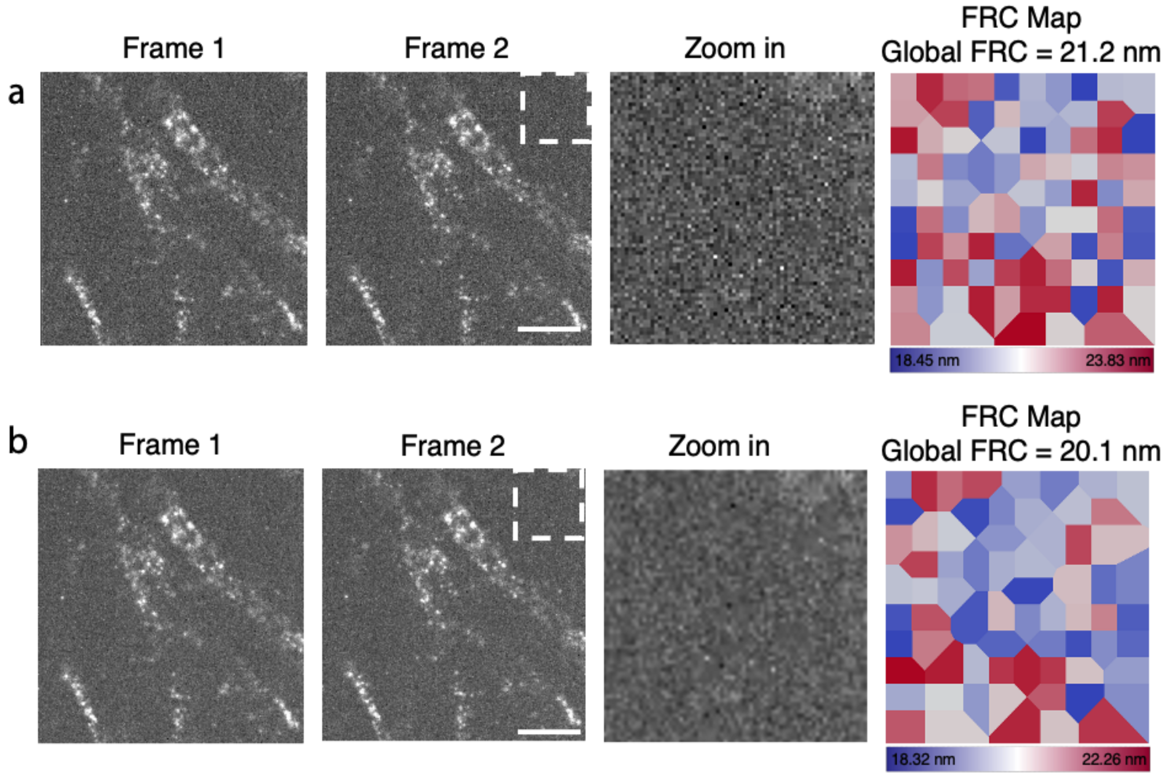

Supplementary Figure 1. FRC Comparison.

(**a**) “Frame 1” and “Frame 2”: two independent confocal images (single xy-plane) of expanded HEK293 cells with pre-expansion microtubule staining in the same region of interest (ROI) under the same imaging conditions, for noise realization. “Zoom in”: Zoomed-in image of the white dotted box in “Frame 2.” Right: Local mapping of FRC resolution values. Each block shows the local FRC resolution value. A global FRC resolution value is calculated by averaging FRC resolution values of all blocks. (**b**) “Frame 1” and “Frame 2”: two Gaussian-filtered (sigma = 0.5) independent confocal images (single xy-plane) of expanded HEK293 cells with pre-expansion microtubule staining in the same region of interest (ROI) under the same imaging conditions, for noise realization. “Zoom in”: Zoomed-in image of the white dotted box in “Frame 2.” Right: Local mapping of FRC resolution values. Each block shows the local FRC resolution value. A global FRC resolution value is calculated by averaging FRC resolution values of all blocks. Scale bars are provided in biological units (i.e., physical size divided by expansion factor) throughout all figures: (**a**) 500 nm, (**b**) 500 nm.

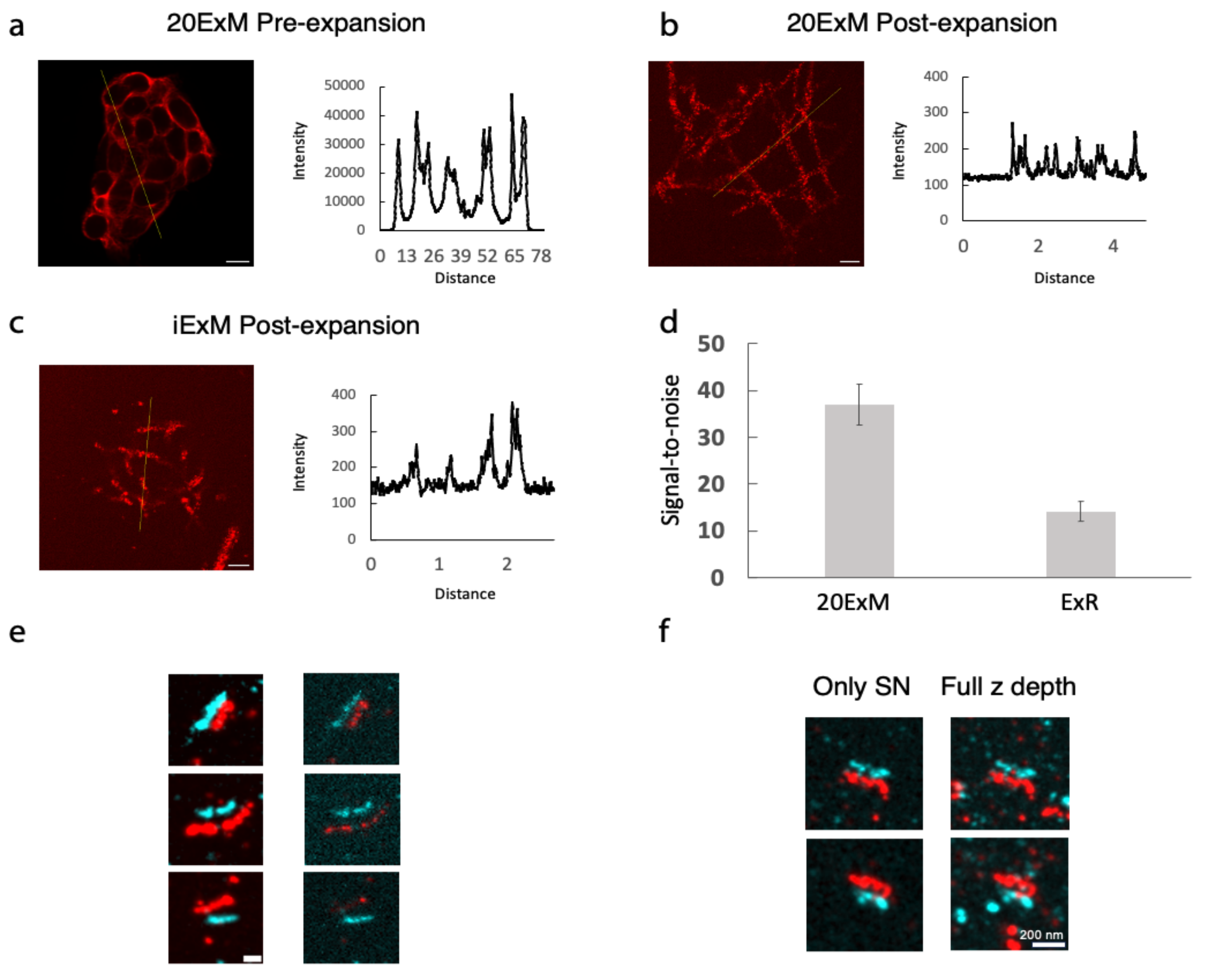

Supplementary Figure 2. Signal Intensity Analysis.
(**a**) Left: Confocal image (single xy-plane; from one representative experiment from three culture batches) of HEK293 cells with pre-expansion microtubule staining. Right: Line intensity profile plot along the yellow line. Brightness and contrast settings: set by Fiji's auto-scaling function. The image was taken with a 40x water immersion objective and 500 ms exposure time. (**b**) Left: Confocal image (single xy-plane; from one representative experiment of three culture batches) of 20ExM-expanded HEK293 cells with pre-expansion microtubule staining. Right: Line intensity profile plot along the yellow line. Brightness and contrast settings: set by Fiji's auto-scaling function. The image was taken with a 40x water immersion objective and 500 ms exposure time. (**c**) Left: Confocal image of iExM-expanded HEK293 cells with pre-expansion microtubule staining. Right: Line intensity profile plot along the yellow line. (Source data: raw data from ref. 8; from 1 expanded sample) Brightness and contrast settings: set by Fiji's auto-scaling function. The image was taken with a 40x water immersion objective and 400 ms exposure time. (**d**) Signal-to-noise ratio analysis of 20ExM RIM1/2 and PSD95 images (n = 90 synapses from 4 brain slices from 2 mice) and ExR Bassoon, Cav2.1, Homer1, PSD95, RIM1/2, Shank3, SynGAP images (n = 3456 synapses from 3 mice). Data are presented as mean values ± standard deviation. We were not able to use the same primary and secondary antibodies that was used for the post-expansion antibody staining ExR protocol because the RIM1/2 primary antibody used in ExR was discontinued. (Source data: Extended Fig. 2d from ref. 10). (**e**) Left column: Zoomed-in max z

intensity projected confocal images of RIM1/2 (red) and PSD95 (cyan) channels, same as Fig. 3b. Brightness and contrast settings: first set by Fiji's auto-scaling function and then manually adjusted to improve contrast and highlight the boundary of synapses. Right column: same images as the left column, but with contrast manually adjusted to have only 1 pixel saturated per channel per image, to highlight the internal heterogeneity within the distribution of RIM1/2 and PSD95. (**f**) Left column: Confocal images (maximum intensity projection over z depths containing the particular synapse) of RIM1/2 and PSD95 signals in 20ExM-expanded mouse brain tissue. Left column: Confocal images (maximum intensity projection over the full imaging depth) of RIM1/2 and PSD95 signals in 20ExM-expanded mouse brain tissue. Quantitative analysis in a–d was conducted on raw image data. Scale bars are provided in biological units (i.e., physical size divided by expansion factor) throughout all figures: (**a**) 10 μm, (**b**) 500 nm, (**c**) 500 nm, (**e**) 100 nm, (**f**) 200 nm.

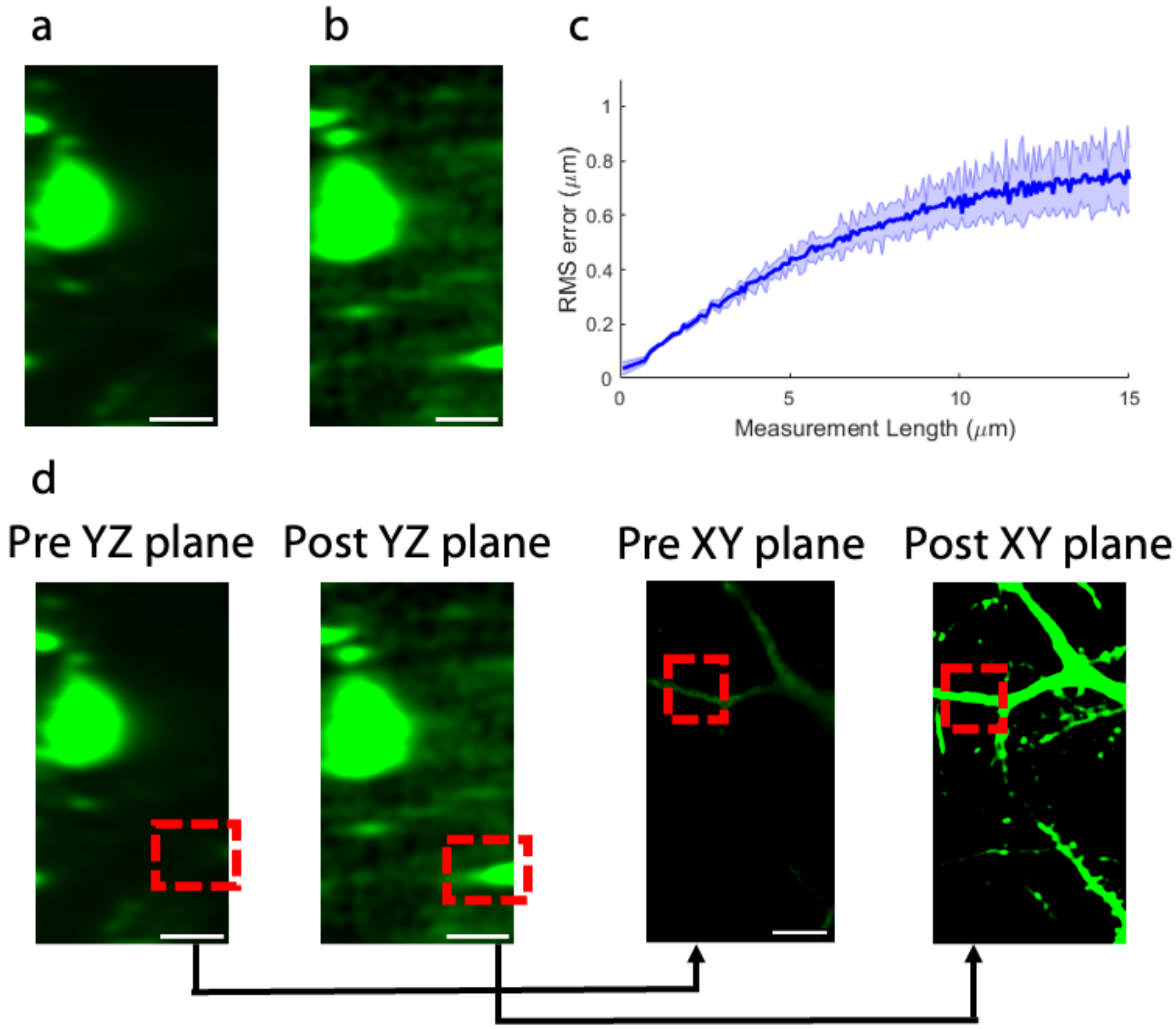

Supplementary Figure 3. Z-axis Distortion Analysis.
(**a**) Pre-expansion 40x-magnification yz-plane confocal image and (**b**) post-expansion 4x-magnification yz-plane Gaussian-filtered (sigma = 4) confocal image in the same Thy1-YFP transgenic mouse brain slice. We made our best attempt to find matching yz-planes in pre- and post-expansion samples. However, the yz-planes still differed slightly, due to differences in sample orientation during confocal imaging before and after expansion. For example, the signal present in **b** near the lower right corner is not visible in **a**, and instead appears in adjacent yz-planes in the pre-expansion sample. Nonetheless, we registered the signals that were present in both images, to quantify distortion. (**c**) Root mean squared (RMS) measurement error as a function of measurement length, comparing pre-expansion 40x-magnification confocal images of Thy1-YFP transgenic mouse brain to post-expansion 4x-magnification images of same regions (blue line, mean; shaded area, ±1 standard deviation; n = 3 areas from two brain slices from one mouse). We used the 4x objective for expanded samples to ensure the field of view of **a** and **b** was as similar as possible, for downstream registration. (**d**) We noticed that signals in pre-expansion images were dim at high-z positions (i.e., further away from the imaging lens;

right side of “YZ plane” images, such as in **a**). This is because we use thick brain slices (50 μm thick), relative to the performance of a confocal microscope, and scattering of light by lipids resulted in decreased signal intensity at depth, whereas expanded (and therefore cleared) samples did not suffer from the same signal decrease. This resulted in apparent differences at high-z positions between pre- and post-expansion images. For example, the red-boxed area in the post-20ExM YZ-plane image is highly visible, whereas the boxed area in the pre-20ExM image only contains a faint signal (left two panels). That is because this boxed area is at a deep z-depth, as far as confocal imaging is concerned. When we examined the same area in the XY-plane image at this z position, we observe the same neuron in pre and post-20ExM images (right two panels). Consistent with lipid scattering, in the “Pre XY plane” image, there is less signal than in the “Post XY plane” image. **Left two panels**: pre- and post-expansion YZ-plane images of the same sample at corresponding X positions. Towards the right, is deeper in the slice (farther from the objective lens of the confocal). **Right two panels**: pre- and post-expansion XY-plane images of the same sample at corresponding Z positions. Red-dotted boxes mark approximately the same region in all images. The “Pre YZ plane” and “Pre XY plane” images have the same brightness and contrast settings. The “Post YZ plane” and “Post XY plane” have the same brightness and contrast settings. Scale bars are provided in biological units (i.e., physical size divided by expansion factor): (**a**) 10 μm, (**b**) 10 μm, (**d**) 10 μm.

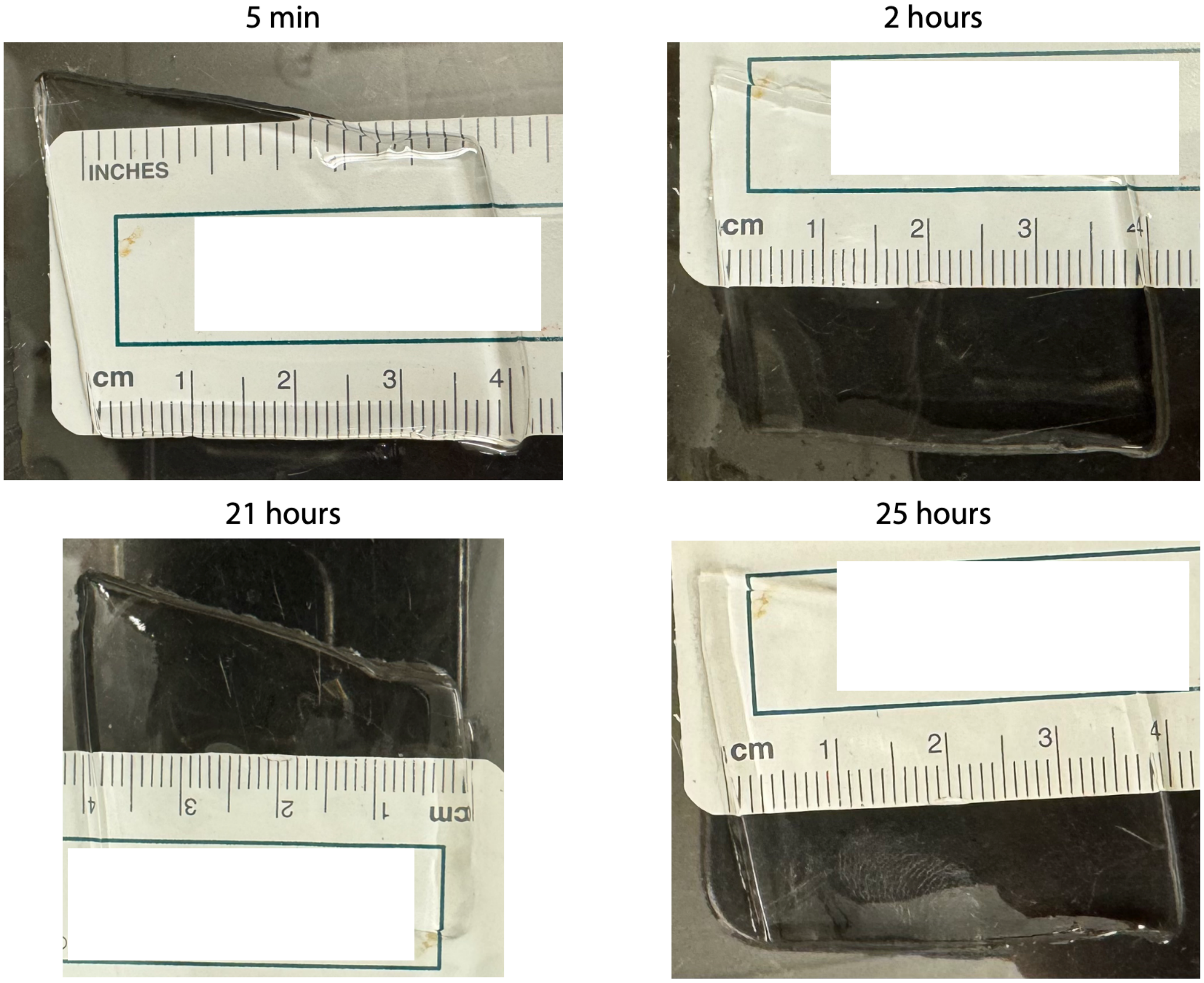

Supplementary Figure 4. Stability of expanded gels.
A gel at various time points (5 minutes, 2 hours, 21 hours, 25 hours) after it reached full expansion. The gel was kept within a capped imaging plate not immersed in water in between the time points. Four white rectangles were added to the figure to cover the ruler logo.

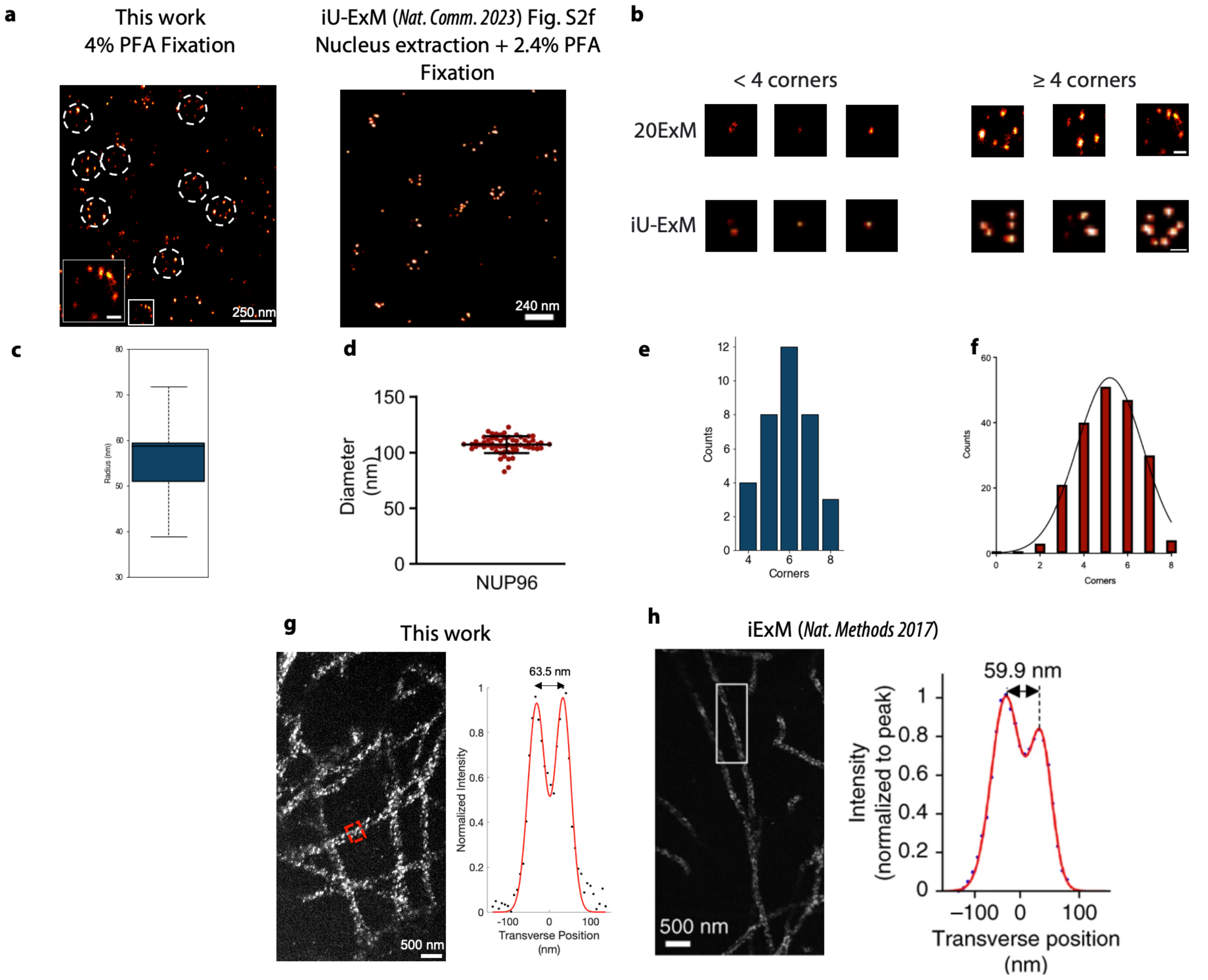

Supplementary Figure 5. Comparison with previous ExM images.

(**a**) **Left**, our work (same as Ext. Fig. 5a); **Right**, state of the art ExM images of nuclear pores with a similar staining strategy (iU-ExM, ref 20, their Fig. S2f). (**b**) **Top row**, selected from our work, for nuclear pores with less than 4 corners visible (left) and with more than or equal to 4 corners visible (right); **Bottom row**, nuclear pores from a state of the art ExM study with a similar staining strategy, for nuclear pores with less than 4 corners visible (left) and more than or equal to 4 corners visible (right). (**c**) Our work: a box plot of NPC radius (same as Ext. Fig. 5b). (**d**) The aforementioned state of the art nuclear pore ExM study: a scatter plot of NPC diameter (iU-ExM, ref 20, their Fig. 2i). (**e**) Our work: a histogram of the number of corners visible per NPC (same as Ext. Fig. 5c). (**f**) From the aforementioned state of the art nuclear pore ExM study: a histogram of number of corners visible per NPC (iU-ExM, their Fig. S2i). (**g**) **Left,** Microtubule images from our work (part of Fig. 2a, imaged in the middle of a cell, with microtubules entering and exiting the imaging plane). **Right**, transverse profile of a microtubule cross-section, indicating a peak-to-peak distance of 63.5 nm (same as Fig. 2b). (**h**) **Left**, state of the art microtubule ExM image. (iExM, ref 8, their Fig. 2d; imaged at the bottom of a cell; this leads to the appearance of longer microtubule segments, since they are flat and parallel to the bottom of the cell, and thus run for longer distances in the imaging plane). **Right**, transverse profile of a microtubule cross-section, indicating a peak-to-peak distance of 59.9 nm (iExM, their Fig. 2e). Scale bars are provided in

2e). Scale bars are provided in biological units (i.e., physical size divided by expansion factor) (**a**): Left, 250 nm; inset, 50 nm; Right, 240 nm. (**b**), 50 nm. (**g**), 500 nm. (**h**), 500 nm.

Supplementary Table 1. High-resolution ExM comparison

	Resolution									Protocol				
Protocol	*Claimed expansion factor*	*Effective expansion factor of cell culture*	*Can fully expand mouse brain?*	*Claimed resolution with conventional microscopy*	*Measured resolution on a conventional microscope*	*How is this resolution measured?*	*Is microtubule imaged with pre-expansion antibody staining?*	*Is hollow structure observed with conventional microscope?*	*What is the average peak-to-peak distance?*	*# of anchoring steps*	*# of gelations*	*Method of digestion for cell culture*	*Method of digestion for mouse brain*	*Post-expansion staining of proteins?*
20ExM	20	21.5	Yes, 18	18 nm	17.9 nm	Fourier-ring correlation	Yes (Fig 2)	Yes	62.1 nm	1	1	LysC/Trypsin; 37˚C, 12-16 hrs	SDS, DTT; 95˚C, 1 hr	Yes
Magnify	11	9.22	Yes, 11	25 nm	N/A	N/A	Yes (Fig 5, S4)	No	N/A	0	1	SDS, Urea, EDTA; 80˚C, 6 hrs	SDS, Urea, EDTA; 80˚C, 8 hrs	Yes
TREx	10	9.4	Yes, 10	Not claimed	N/A	N/A	Yes (Fig 3)	Yes	No population data	1	1	ProK; 37˚C, 4 hrs	ProK; rt, 3 hrs + SDS; 80˚C, 3 hrs	No
X10	10	11.5	Yes, 9.6	25-30 nm	25.2 nm	Full width at half maximum	Yes (Fig 2)	No	N/A	1	1	ProK; 50˚C, >12 hrs	ProK; 50˚C, >12 hrs	No
X10ht	10	10	No, only 6	20-25 nm	N/A	N/A	Yes (Fig 1)	No	N/A	1	1	SDS, Triton-X; rt, 2 hrs + 121˚C, 30 min	SDS, Triton-X; rt, 2 hrs + 121˚C, 30 min	Yes
ExR	20	7.7-8, neuron culture	Yes, 20	20 nm	17.6 nm	Distance between two distinct signals	No	N/A	N/A	0	3	SDS; 95˚C, 1 hr	SDS; 95˚C, 1 hr	Yes

Pan-ExM-t	24	15.7	Yes, 24.1	Not claimed	N/A	N/A	No	No	N/A	1	3	SDS; 73˚C, 1 hr	SDS; 75˚C, 4 hr	Yes
iExM	20	16-22	Yes, 20	25 nm	25.8 nm	Full width at half maximum	Yes (Fig 2)	Yes	58.7 nm	1	3	ProK; rt, >12 hrs	ProK; rt, >12 hrs	No
iU-ExM	16-22	14-26	Did not test brain	10-20 nm	20 nm	Distance between two distinct signals	No	N/A	N/A	1	3	SDS; 85 ˚C, 1.5 hrs	N/A	Yes

Supplementary Table 2. Measured expansion factor of cell culture and mouse brain tissue based on biological landmarks

Cell culture							Brain tissue				
Replicate	Cell	Measurement	Pre-dist (µm)	Post-dist (µm)	ExpFactor	Average per replicate	Replicate	Measurement	Pre (µm)	Post (µm)	ExpFactor
1	1	1	18.14	385.13	21.23		1	1	23.57	426.46	18.09
		2	26.85	552.13	20.56			2	16.18	293.15	18.12
		3	20.32	442.04	21.75			3	13.82	261.48	18.92
	2	1	26.90	451.50	16.78		2	1	10.89	201.03	18.46
		2	32.86	604.34	18.39			2	10.91	200.05	18.34
		3	21.45	376.65	17.56	19.38		3	4.94	92.38	18.70
2	1	1	12.50	270.48	21.64					*Total Avg*	*18.44*
		2	5.68	128.77	22.67					*Total Std*	*0.33*
		3	12.18	263.16	21.61						
	2	1	15.16	322.93	21.30						
		2	22.83	503.10	22.04						
		3	11.17	248.84	22.28	21.92					
3	1	1	18.19	405.77	22.31						
		2	10.55	233.52	22.13						
		3	9.98	225.24	22.57						
	2	1	14.80	330.28	22.32						
		2	17.52	389.29	22.22						
		3	26.16	630.06	24.08	22.61					
4	1	1	14.99	347.65	23.19						
		2	16.40	368.80	22.49						
		3	17.49	381.88	21.83						
	2	1	15.22	339.94	22.34						

		2	15.17	315.20	20.78						
		3	19.62	429.00	21.87	22.08					
				Total Avg	*21.50*						
				Total Std	*1.70*						

Supplementary Table 3. Antibody list

Type	Target	Host	Vendor	Product number	Dilution/Conc.
20ExM Cell Culture					
Primary	Beta tubulin	Rabbit	Abcam	ab6046	1:100
Primary	mNeonGreen	Mouse	Proteintech	32f6	1:100
Primary	TOM20	Rabbit	Proteintech	11802-1-AP	1:100
Secondary	Rabbit	Goat	ThermoFisher	A11035 (Alexa Fluor 546)	1:100
Secondary	Mouse	Goat	ThermoFisher	A21043 (Alexa Fluor 568)	1:100
Tertiary	Goat	Donkey	ThermoFisher	A32816 (Alexa Fluor Plus 555)	1:200
20ExM Brain Tissue					
Primary	RIM1/2	Guinea pig	Synaptic Systems	140205	1:200
Primary	PSD95	Mouse	ThermoFisher	MA1-046	1:200
Primary	GFP	Rabbit	ThermoFisher	A11122	1:200
Secondary	Guinea pig	Goat	ThermoFisher	A21435 (Alexa Fluor 555)	1:200
Secondary	Mouse	Donkey	ThermoFisher	A32787 (Alexa Fluor Plus 647)	1:200
Secondary	Rabbit	Goat	ThermoFisher	A11008 (Alexa Fluor 488)	1:200

Supplementary Note 1: Protocol of 20ExM

Biological Samples

- Cultured cells: fixed (4% paraformaldehyde or 3% paraformaldehyde/0.1% glutaraldehyde (the choice of fixative would be at the discretion of the user; please choose based upon what your experience or expertise suggests), in phosphate buffered saline (PBS)), primary and fluorescent or nonfluorescent (a fluorescent tertiary will be used after softening to boost signal intensity) secondary immunostained, AX-treated cell culture (see below for details on preparation). In practice, AX could be replaced by GMA (the epoxide anchor used in *PLoS One* **2023**, *18* (9), e0291506); while we do not anticipate any issues with such a substitution, please be aware we have not formally validated the expansion factor and isotropy of 20ExM when GMA is used.
- Brain: fixed (4% paraformaldehyde in PBS), 50-µm thick, AX-treated, microdissected mouse brain slices
- Spleen or kidney: fixed (4% paraformaldehyde in PBS), 50-µm thick, microdissected, primary and secondary immunostained, AX-treated spleen or kidney slices

  Note 1: 20ExM, in the form presented here, has only been validated with post-expansion antibody staining for the case of brain tissue, and not other kinds of specimen (because standard SDS softening works well for hydrogel-embedded brain tissue, but not many other sample types). 20ExM has been validated with pre-expansion antibody staining of cell cultures, and kidney and spleen tissues (because pre-expansion staining is compatible with strong protease softening, appropriate for these kinds of specimens). 20ExM also supports post-expansion NHS pan-protein staining for cell cultures, and brain, kidney, and spleen tissues (because such staining works on the proteolyzed fragments that remain after proteinase treatment).

  Note 2: The secondary antibody used in pre-expansion staining can be fluorescent or non-fluorescent. In both cases, tertiary fluorescent antibodies will be added to boost fluorescence further. Multi-color antibody staining can be achieved even with tertiary staining, as long as orthogonal antibody-species sets are available. For example, to use two primary antibodies simultaneously, users can use 6 antibodies of different species and/or subtypes, comprising 2 antibody sets, such as (rabbit primary, chicken anti-rabbit secondary, and fluorescent donkey anti-chicken tertiary), and (mouse primary, rat anti-mouse secondary, and fluorescent goat anti-rat tertiary).

  Note 3: AX (N-acryloxysuccinimide) performs the same anchoring functions as AcX (Acryloyl-X, SE), as used in earlier ExM protocols such as proExM, but is cheaper. We use AX for all samples in 20ExM. In summary:

  - *Cultured cells: all cells are incubated in AX solution (N-acryloxysuccinimide; Thermo Scientific, catalog no. 400300010; dilution of 10 mg/mL DMSO stock in 1× PBS, 1:2000, 60 µL per well for 16-well chambered coverglass or 300 µL per well for 24-well glass-bottom plates) at room temperature (~24 ˚C) overnight*

(12–20 hours). Then, they are washed in 1× PBS for 10 minutes at room temperature (~24 ˚C).

  - *<u>Brain:</u> each brain slice is incubated in AX solution (N-acryloxysuccinimide; Thermo Scientific, catalog no. 400300010; dilution of 10 mg/mL DMSO stock in 100 mM MES, 150 mM NaCl pH 6 buffer, 1:200, 1 mL) at 4 ˚C overnight (12–20 hours). The slices are then washed with 1 mL 1× PBS for 10 minutes at room temperature (~24 ˚C).*
  - *<u>Kidney and spleen:</u> slices are microdissected into ~1 mm × 1 mm subregions and immunostained with primary and secondary antibody (if desired). (Kidney and spleen slices are microdissected before antibody staining and AX treatment because their sizes are large, meaning wasted reagents if regions are not going to be imaged.) Each section is incubated in AX solution (N-acryloxysuccinimide; Thermo Scientific, catalog no. 400300010; dilution of 10 mg/mL DMSO stock in 100 mM MES, 150 mM NaCl pH 6 buffer, 1:200, 50 µL) at 4 ˚C overnight (12–20 hours). The section is then washed with 50 µL 1× PBS for 10 minutes at room temperature (~24 ˚C).*

Key materials and tools

- 16-well chambered coverglasses (Grace Bio-Labs, catalog no. 112359)
- Coverglass removal tool (Grace Bio-Lab, catalog no. 103259)
- Glove bag (GlasCol, catalog no.108D X-17-17HG)
- Compressed nitrogen cylinders
- 20-mL glass vials (ChemGlass, catalog no.CG-4908-03)
- Gas dispersion tube (ChemGlass, catalog no. CG-203-04)
- Platform (e.g., a plate lid of a 6-well or 24-well plate)
- Airtight chamber (e.g., Rubbermaid Brilliance Food Storage Containers)
- Glass slides and coverslips
- Tweezer
- P1000, P200, P20, P10 pipets and pipet tips
- Transfer pipet
- 1.5-mL centrifuge tubes
- Paint brush
- 6-well glass-bottom plates (Cellvis, catalog no. P06-1.5H-N)
- Imaging plates (MatTek, catalog no. P384G-1.5-10872-C)

Key chemicals and buffers

- Trichloro(octadecyl)silane (Fisher Scientific, catalog no. AC147400250)
- Hexane (Sigma, catalog no. 296090)
- Acidified Tris buffer (10% (v/v) 1 M Tris-HCl pH 8 buffer, 20% (v/v) 1.2 M HCl in ddH_2O)
- Sodium acrylate (AK Scientific, catalog no. R624)
  Note: Sodium acrylate quality varies between vendors and batches. High-quality sodium acrylate is required. As noted at expansionmicroscopy.org: "Sodium acrylate batches from different vendors, or from different lots, can vary in quality. Low-quality sodium

acrylate may not completely dissolve in water at the relatively high concentration used in ExM, or may appear yellow or orange when dissolved in water. If the sodium acrylate solution is cloudy, or appears yellow or orange, discard the solution and switch to a new bottle of sodium acrylate." For 20ExM, clear solutions of sodium acrylate are required. The gelation solution should be colorless and non-cloudy. We recommend using the same vendor as reported in this manuscript, at least at time of writing this protocol, and always recommend verifying the quality through aforementioned checks. Updates on recommended vendors will be posted periodically at expansionmicroscopy.org.

- TEMED (tetramethylethylenediamine; Sigma, catalog no. T7024)
- DMAA (N,N-dimethylacrylamide; Sigma, catalog no. 274135)
  Note: DMAA and TEMED should be kept in a dark, dry environment and should be replaced every three months.
- Potassium persulfate (Sigma, catalog no. 379824)
- LysC/Trypsin protease (ThermoFisher, catalog no. A41007)
- 100 mM Tris-HCl pH 8 buffer
- DTT (dithiothreitol; Sigma, catalog no. D9779)
- Denaturation buffer (5% (v/v) sodium dodecyl sulfate (SDS), 200 mM NaCl, 50 mM Tris pH 8)
- 1× PBS (phosphate buffered saline)
- Triton X-100 (Sigma, catalog no. X100)
- Normal donkey serum (NDS; Jackson ImmunoResearch, catalog no. 017-000-121)

Manufacture hydrophobic glass slides and coverslips

Steps 1-4 are performed in a chemical fume hood with proper PPE at room temperature (~24 ˚C).

1. Add 20 µL trichloro(octadecyl)silane to 10 mL hexane.
2. Immerse glass slides and coverslips in the solution for 90 seconds.
3. Remove glass slides and coverslips from the solution with a tweezer.
4. Rinse the glass with 70% isopropanol and ddH_2O sequentially.
5. Place glass inside a 37˚C incubator to dry.
6. Wipe off white residual reactants (expected) with a dry kimwipe.
   Note: Hydrophobic glass slides and coverslips can, if properly handled, be reused at least 15 times. After each use, wash hydrophobic glass with ddH_2O and gently wipe with kimwipe. These steps do not need to be repeated for every gelation.

Set up glove bag

1. Place the glove bag on a bench.
2. Connect the glove bag to a tube attached to a compressed nitrogen cylinder nearby.
3. Seal the connection between the tube and the glove bag with tape (Extended Fig. 2b).
4. Fill the glove bag with nitrogen. Then turn off the nitrogen and observe if the glove bag is slowly deflating. If that is the case, the glove bag is not airtight, and a small flow of nitrogen can be provided to keep the bag inflated.

Gelation

1. Gelation solution: dissolve 0.522 g sodium acrylate in 1 mL acidified Tris buffer in a 20-mL glass vial. Vortex.
2. Add 10 µL TEMED to 90 µL ddH_2O in a 1.5-mL centrifuge tube. Vortex.
3. Add 7.5 µL 10% TEMED solution to the 20-mL glass vial.
4. Add 900 µL DMAA to the 20-mL glass vial. Vortex.
   Note 1: DMAA and subsequent gelation solution are viscous. To ensure accurate volume, pre-wetting the pipet tip is required.
   Note 2: The gelation solution should be colorless and non-cloudy. Otherwise the sodium acrylate and DMAA quality is low or has degraded.
5. Initiator solution: dissolve 45 mg potassium persulfate in 1 mL ddH_2O in a 1.5-mL centrifuge tube to make the initiator solution. Vortex for 2 minutes.
   Note: potassium persulfate takes time to fully dissolve and will precipitate from the solution if placed on ice. Do not place initiator solution on ice.
6. Place the gelation solution on ice.
7. Construct the airtight humidified chamber: place a damp towel in the bottom of the airtight chamber. Place a platform on top of the damp towel (Extended Fig. 3g).
8. Construct the gelation chamber: wrap parafilm strips with size ~4.5 cm × 0.2 cm around the hydrophobic glass slide with 0.4-cm gap for cell culture or 0.1-cm gap for brain tissue (Extended Fig. 3a,e).
   Note 1: For cells grown in Grace BioLabs 16-well chambered coverglasses, use the coverglass removal tool to remove the upper part of the chamber (Extended Fig. 3d). Then use a pair of tweezers to carefully remove the remaining rubber on top of the coverglass (Extended Fig. 3e). For cells grown in 24-well plates on top of glass coverslips, use techniques described in Fig. 3 of Curr. Protoc. Neurosci. ***2020****, 92 (1), e96 to lift up the coverslip and construct the gelation chamber. Use No. 2 glass coverslips to avoid breaking the coverslips.*
   Note 2: The tissue that we use is approximately the size of the dotted white box in Fig. 3a and is 50-µm thick, which can fit within the 0.1-cm gap. Using larger tissue is possible but the large size after 20-fold expansion may make the gel difficult to fit into the imaging plate and onto the confocal microscope. We do not recommend the current protocol, as it stands, for tissue thicker than 100 µm.
9. Transfer biological specimen into the gelation chamber (Extended Fig. 3b,e). The biological specimen, for the purposes of this protocol, is:
   a. a cell culture that has been fixed with 4% paraformaldehyde or 3% paraformaldehyde/0.1% glutaraldehyde, stained with primary and secondary antibodies, and treated with AX, or
   b. a brain tissue slice that has been fixed with 4% paraformaldehyde, treated with AX, and microdissected, or

c. a kidney or spleen tissue slice that has been fixed with 4% paraformaldehyde, microdissected, stained with primary and secondary antibodies (if needed), and treated with AX

Note: biological specimens are immersed in 1× PBS to avoid dehydration. Use a paint brush to transfer tissues.

10. Place the gelation chambers containing biological specimens into the airtight humidified chamber (Extended Fig. 3g,h).
11. In a chemical fume hood, connect gas dispersion tube to a compressed nitrogen cylinder.
    Note 1: The nitrogen flow needs to be kept minimal. Otherwise, the gelation solution will evaporate rapidly and freeze. For first-time users, please practice controlling the nitrogen flow in a 20-mL glass vial filled with 5 mL water to determine the minimal nitrogen flow required to generate bubbles.
    Note 2: The sponge head of the gas dispersion tube needs to be fully wetted to generate bubbles.
12. Immerse a clean, dry gas dispersion tube with flowing nitrogen in gelation solution for 50 seconds in a chemical fume hood (Extended Fig. 2a).
13. Cap the vial quickly after removing the gas dispersion tube to minimize oxygen exposure.
    Note: The 20-mL glass vial is not airtight. Minimize the time between the completion of oxygen removal and placing the vial in the nitrogen-filled glove bag.
14. Move pipets (P1000, P200, P20), pipet tips, transfer pipets, a tweezer, airtight humidified chamber with gelation chambers, two 1.5-mL centrifuge tubes, gelation solution, initiator solution, and hydrophobic glass coverslips into the glove bag.
    Note 1: No ice or cold block is needed. All subsequent steps are performed at room temperature. The tissue gelation protocol has been optimized to not gelate for at least 45 minutes upon the addition of initiator solution.
    Note 2: Putting pipet tips onto pipets before moving them into the glove bag can reduce tasks performed inside the glove bag.
15. Turn on the nitrogen flow. Purge the glove bag by filling the glove bag with nitrogen then pushing on top to remove most of the gas within. Repeat purging three times.
    Note: Ensure airtight chamber is not capped during purges.
16. Seal the glove bag and turn off the nitrogen flow.
    Steps 17–22 are performed inside the glove bag
17. Inside the glove bag, add 411 µL gelation solution and 20 µL (cell culture) or 4 µL (tissue) initiator solution in a 1.5-mL centrifuge tube. Flip the tube upside down five times for mixing.
18. Remove the PBS immersing biological specimens with a transfer pipet.
19. Add 50 µL activated (i.e., initiator-supplemented) gelation solution to each biological specimen.
20. For cell culture, use a tweezer to flip the coverslip to turn the cell culture facing down to construct the gelation chamber (Extended Fig. 3f).

21. For brain tissue, tissue is incubated in the activated gelation solution for 15 minutes in the sealed airtight humidified chamber. After incubation, use a tweezer to place a hydrophobic glass coverslip on top to cap the gelation chamber (Extended Fig. 3c).
22. Place gelation chambers in the sealed airtight humidified chamber.
23. Remove the airtight humidified chamber from the glove bag. Place it in the dark at room temperature for 2 hours (cell culture) or overnight (16–20 hours, tissue).
24. After incubation, cut out sections of gel containing biological specimens and place sections in a 6-well plate, one section per well. Measure the pre-expansion gel size if needed, as described in Supplementary Note 9, before any washes.
    Note 1: To stop polymerization after cutting, the gel needs to be placed within softening solution (which will start softening immediately), or 5 M NaCl solution (if short-term storage is preferred, before starting softening). Polymerization will continue to proceed until the gel is immersed in softening solution or salt solution. We suggest starting to cut gels early so the gel can be placed in the softening solution as close to the 2-hour mark (for cell culture) or the 16-20 hour mark (for tissue; this window, being broader, may be less demanding to hit) as possible.
    Note 2: For sections containing cell culture, cut the gel in the shape of a trapezoid to ensure that cells are facing down in the expansion step, as described in Fig. 3 of Curr. Protoc. Neurosci. ***2020****, 92 (1), e96.*

Softening

1. For cell culture, dissolve 20 µg LysC/Trypsin protease in 1 mL 100 mM Tris-HCl pH 8 buffer. Incubate one section of gel in 1 mL buffer at 37˚C overnight (12–16 hours). Skip to Step 4. Follow section "Immunostaining for samples with pre-expansion immunostaining".
   Note 1: LysC/Trypsin digestion does not support post-expansion primary and secondary staining of cell culture. We have tested SDS softening on cell cultures fixed by 3% paraformaldehyde and 0.1% glutaraldehyde, and found that SDS softening does not enable isotropic expansion of nanostructures universally, such as microtubules. Thus, we currently recommend performing pre-expansion staining, and then to use LysC/Trypsin digestion for softening, for the purposes of the current 20ExM protocol.
   Note 2: This is not to say that post-expansion staining for cell culture is impossible - we simply have not tested if SDS softening can enable isotropic expansion for cells fixed by 4% paraformaldehyde, yet, as we note that some ultrastructural components, such as microtubules and mitochondria, do require 3% paraformaldehyde and 0.1% glutaraldehyde fixation to be preserved (Science ***2007****, 317 (5845), 1749–1753; Nat. Methods* ***2008****, 5 (12), 1047–1052). Thus, it is possible that post-expansion staining of cell culture will require further optimization, especially if a universal protocol is desired.*
2. For brain tissue, dissolve 10 mg DTT in 1 mL Denaturation buffer. Incubate one section of gel in the buffer at 95˚C for 1 hour. Skip to Step 4. Follow section "Immunostaining for samples without pre-expansion immunostaining".

3. For kidney and spleen tissue, dissolve 20 µg LysC/Trypsin protease in 1 mL digestion buffer (1 mM EDTA, 50 mM Tris-HCl pH 8 and 0.1 M NaCl). Incubate one section of gel in 1 mL buffer at 37˚C overnight (12–16 hours). If pre-expansion primary and secondary antibody staining was performed, follow section "Immunostaining for samples with pre-expansion immunostaining". Otherwise, follow NHS staining protocol: Gels containing kidney or spleen tissue were incubated in NHS staining solution (Alexa Fluor 488 NHS Ester; Thermo Scientific, catalog no. A20000; dilution of 10 mg/mL DMSO stock in 1× PBS, 1:50, 1 mL) at 4 ˚C overnight (12–24 hours) and washed in 1× PBS three times, 20 minutes each, on a shaker at 40 rpm at room temperature (~24 ˚C).
   Note 1: LysC/Trypsin digestion does not support post-expansion primary and secondary staining of kidney and spleen tissue. And as noted, our current SDS softening protocol does not guarantee isotropic expansion of such fibrous tissues. The current protocol supports post-expansion NHS pan-protein staining or pre-expansion antibody staining for kidney and spleen tissues.
   Note 2: For antibody staining, we recommend performing primary and secondary antibody staining before anchoring, as described in the proExM protocol (Nat. Biotechnol. ***2016****, 34 (9), 987–992), and follow "Immunostaining for samples with pre-expansion immunostaining" section to boost signal intensity.*
4. Wash softened gels in 1× PBS twice for 15 minutes.

Immunostaining for samples with pre-expansion immunostaining

Note: Follow this section for samples with pre-expansion primary and secondary antibody staining such as cell culture and kidney/spleen tissue. The goal of this section is to boost signal intensity by applying a fluorescent tertiary antibody against the secondary antibody (fluorescent or non-fluorescent) used pre-expansion, or applying a fluorescent secondary antibody against the primary antibody used pre-expansion if tertiary antibody is not available. For conciseness, we will refer to tertiary or secondary antibody applied in this section as "boost antibody."

1. Incubate sections of gel in 200 µL blocking solution (0.5% Triton X-100, 5% normal donkey serum (NDS; this should be whatever the species in which the boost antibody was raised) in 1× PBS) for 2 hours at room temperature (~24 ˚C). Tilt the plate to ensure gels are fully immersed and add more blocking solution if necessary.
2. Aspirate blocking solution and incubate sections of gel in 200 µL staining solution (0.25% Triton X-100, 5% NDS in 1× PBS) containing boost antibody (typically 1:200 dilution of 1 mg/mL antibody solution; see Supplementary Table 3 for antibody concentrations used in this manuscript) overnight (12–24 hours) at 4 ˚C. Tilt the plate to ensure gels are fully immersed and add more staining solution if necessary.
3. Aspirate solution and incubate sections of gel in washing solution (0.1% Triton X-100 in 1× PBS) for 30 minutes on a shaker at 40 rpm at room temperature (~24 ˚C). Repeat the washing 3 more times.

Immunostaining for samples without pre-expansion immunostaining

Note: Follow this section for samples without pre-expansion primary and secondary antibody staining such as brain tissues. The goal of section is to perform primary and secondary antibody staining to label biomolecules after softening.

1. Incubate sections of gel in 200 µL blocking solution (0.5% Triton X-100, 5% normal donkey serum (NDS; this should be whatever the species in which the secondary antibodies were raised) in 1× PBS) for 2 hours at room temperature (~24 ˚C). Tilt the plate to ensure gels are fully immersed and add more blocking solution if necessary.
2. Aspirate blocking solution and incubate sections of gel in 200 µL staining solution (0.25% Triton X-100, 5% NDS in 1× PBS) containing primary antibodies (typically 1:200 dilution of 1 mg/mL antibody solution; see Supplementary Table 3 for antibody concentrations used in this manuscript) overnight (12–24 hours) at 4 ˚C. Tilt the plate to ensure gels are fully immersed and add more staining solution if necessary.
3. Aspirate solution and incubate sections of gel in washing solution (0.1% Triton X-100 in 1× PBS) for 30 minutes on a shaker at 40 rpm at room temperature (~24 ˚C). Repeat the washing for 3 more times.
4. Repeat Steps 2 and 3 for secondary antibody staining.

Expansion

1. To expand, transfer a section of gel from 6-well plate to an imaging plate containing 1× PBS. For sections containing cell culture, ensure the right-trapezoid (i.e., a trapezoid with one side with 90 degree angles with respect to both bases)-shaped gel is in the orientation with cells on the bottom of the gel.
   Note: To gently transfer or flip gels, we suggest using specific strategies, as described in Curr. Protoc. Cell Biol. ***2018****, 80 (1), e56.*
2. Aspirate the solution carefully and slowly add at least 10 mL double distilled water. Incubate the gel for 20 minutes without shaking at room temperature. Repeat this step 2–4 times until the gel no longer expands.
   Note 1: Shining a flashlight from underneath can help visualize gels while aspirating and avoid accidentally damaging the gel.
   Note 2: If additional transferring or flipping the gel is needed, shrink the gel by incubating in 1× PBS for 20 minutes first, which results in a shrinkage to ~4x. Do not transfer or flip the gel when it is fully expanded. Transfer or flip the gel. Then re-expand in water (which will take another hour).
3. Upon full expansion, remove excess water around the gel with transfer pipets and kimwipes. Transport the imaging plate to the microscope for imaging.

Supplementary Note 2: Expansion Factor Measurements

To assess whether physical gel size measurements for both cells and tissues align with the expansion factors observed using biological landmarks, we performed 20ExM on cells and tissues, using our standard protocols for each, to quantitatively measure the expansion factor,

both by assessing physical gel size as well as by examining biological landmarks in pre- vs. post-expansion samples.

For cell culture, we provide physical gel size measurements for two of the four HEK293 cell batches stained with anti-beta-tubulin antibodies and then expanded, that we used for expansion factor characterization (we did not measure physical gel size for the other two cell batches). We measured the size of each gel with a ruler immediately after gelation, and then again after full expansion. We observed 22.4 ± 1.0 (mean ± standard deviation)-fold expansion when physical gel size was assessed, vs. 22.3 ± 0.8-fold expansion when biological landmarks were utilized, for these two cell culture batches (Supplementary Table 2, Supplementary Data).

For tissue, we performed 20ExM with Thy1-YFP transgenic mouse brain slices, using the tissue protocol. Pre-expansion, in order to use biological landmarks to calculate expansion factor, we imaged YFP signals with confocal microscopy before gelation; to measure physical gel size, we used a ruler, after gelation. Post-expansion (in more detail: we performed softening, anti-GFP staining, and expansion), we re-measured the physical size of the gel with a ruler, and re-imaged the same region in the brain slice with a confocal microscope to use biological landmarks to calculate expansion factor (n = 2 brain slices from 1 Thy1-YFP transgenic mouse). We observed 18.5 ± 1.1 (mean ± standard deviation)-fold expansion when physical gel size was assessed, and 19.0 ± 0.7-fold expansion when biological landmarks were utilized (Supplementary Table 2, Supplementary Data).

Thus, the physical gel size-assessed expansion factor for both cells and tissues matched the expansion factor assessed when biological landmarks were used.

We quantitatively measured the z-axis expansion factor of gel-embedded brain tissue (the same specimens used for xy-plane expansion factor measurements described above) by measuring physical gel size, as well as by utilizing biological landmarks, pre- vs post-expansion, with a focus on the z-axis.

For physical gel size, we measured pre- and post-expansion gel thickness with a confocal microscope, and obtained the expansion factor. In particular, we focused on the gel-adjacent surfaces of parafilm spacers (which flank the gel closely and were more autofluorescent, and thus more visible by the confocal, than the gel itself), to determine pre-expansion thickness, and on the expanded gel top and bottom, to determine post-expansion thickness. We observed 18.0 ± 0.4 (mean ± standard deviation; n = 2 brain slices from 1 Thy1-YFP transgenic mouse)-fold z-axis expansion, as assessed by physical gel size (Supplementary Data).

We measured pre- and post-expansion distances between the highest and lowest (along the z-axis) visible YFP signals in the slice (we used 50-µm thick Thy1-YFP brain specimens), to serve as biological landmarks, to calculate expansion factor. We observed 18.2 ± 0.5-fold z-axis

expansion via analysis of these biological landmark signals (mean ± standard deviation; n = 2 brain slices from 1 Thy1-YFP transgenic mouse; Supplementary Data).

These z-axis expansion factors are consistent across the two different methods of measurement, and are also consistent with the xy-plane expansion factors measured above for a gel specimen containing tissue (~18-fold).

Supplementary Note 3: Effect of Gelation Time

To demonstrate how gelation time influences expansion factor and its reproducibility, in the context of tissue expansion, we used Thy1-YFP transgenic mouse brain slices, and performed 20ExM, in brain tissue protocol form, with varying gelation times (6, 16–20 (standard), and 72 hours; n = 2 brain slices from 1 mouse for each condition). We found that 6 hours was not sufficient to complete gelation (i.e., the gel didn't fully polymerize), whereas 16–20-hour gelation samples expanded 18-fold (the same samples referred to, in Supplementary Note 2). The 72-hour gelation samples expanded 10.1 ± 0.3 (mean ± standard deviation)-fold (Supplementary Data).

Supplementary Note 4: Stability of Expanded Gels

We examined the size of an expanded brain-tissue-embedded gel at 5 minutes, and at 2, 21, and 25 hours, after full expansion was achieved, within a capped imaging plate (n = 1 gel). We found that the gel did not visibly contract or exhibit other obvious changes over the course of 25 hours (Supp. Fig. 4). In addition, in all the aforementioned studies relating to expansion factor, consistent expansion factors, with small standard deviations, were observed, without particular attention to timing. Thus, especially in a humidity-controlled environment, gels may be stable over the course of a day or so.

Supplementary Note 5: Expected Microtubule Diameter

Antibody-labeled microtubules have been extensively imaged and commonly used as a standard by the super-resolution community, having been imaged with STORM, STED, ExM, and many other methods (e.g., *Science* **2007**, *317* (5845), 1749–1753; *Nanoscale* **2018**, *10* (37), 17552–17556). For example, in our previous study on iterative expansion with 20x magnification (iExM, *Nat. Methods* **2017**, *14* (6), 593–599), STORM images of primary antibody-stained microtubules resulted in a width that ranged from 25 to 50 nm, with a mean and standard deviation of 37.3 nm and 4.7 nm respectively. The iExM images of primary and secondary antibody (bearing DNA oligos for amplification of brightness)-stained microtubules resulted in a range of 25 to 90 nm, with a mean and standard deviation of 58.7 and 10.3 nm respectively. The latter number, in particular, could be regarded as an estimate of the upper bound (because it includes any real biological variability in microtubule thickness) on the nanoscale error introduced by iExM. It has been modeled and observed that primary antibody-labeled

microtubules have an average diameter around 40 nm, and primary and secondary antibody-labeled microtubules have an average diameter around 60 nm (*Science* **2007**, *317* (5845), 1749–1753; iExM, *Nat. Methods* **2017**, *14* (6), 593–599; *Nanoscale* **2018**, *10* (37), 17552–17556; *EMBO Rep.* **2018**, *19* (9). https://doi.org/10.15252/embr.201845836). Our measurement of 100 microtubule diameters resulted in an average of 62.7 nm and standard deviation of 8.8 nm, which matches the previous iExM result, suggesting a high resolution and low distortion of 20ExM.

See Supp. Fig. 5 for visual comparisons of the current work with prior iExM images. Please note that microtubules in the iExM paper were imaged at the bottom of the cell; this leads to the appearance of longer microtubule segments, since they are flat and parallel to the bottom of the cell, and thus run for longer distances in the imaging plane. Our microtubules are imaged in the middle of the cell, and thus enter and exit the imaging plane.

Supplementary Note 6: Signal Intensity Analysis

We conducted line intensity profile analyses on microtubule images generated using 20ExM or iExM protocols, using previously published iExM data (since the original iExM protocol is not in much use anymore, with the ExR protocol having largely replaced it). Our findings revealed that the 20ExM protocol yielded similar-appearing images, and line profiles, between 20ExM and iExM (Supp. Fig. 2b,c).

In principle, noise in confocal images could originate from multiple sources, including the immunohistochemistry protocol itself (e.g., non-specific binding), and imaging shot noise. We attempted to keep staining protocol noise as small as possible, by strictly following a microtubule staining protocol (i.e., extraction, fixation, etc.) used in previous studies for measuring resolution (*Science* **2007**, *317* (5845), 1749–1753; *Nat. Methods* **2017**, *14* (6), 593–599). To assess the impact of shot noise, we performed FRC analysis on the same image pair both with and without Gaussian filtering, which reduces shot noise (*IEEE Trans. Biomed. Eng.* **2000**, *47* (12), 1600–1609; *Int. J. Biochem. Cell Biol.* **2021**, *140*, 106077). We used a sigma value of 0.5 for the Gaussian filter to avoid blurring the signal too much, which may affect the resolution. Such Gaussian filtering reduces noise visibly, but the Global FRC barely changed – from 21.2 nm for non-Gaussian-filtered images, to 20.1 nm for Gaussian-filtered images (Supp. Fig. 1). Thus our FRC measurements for 20ExM remained consistent, regardless of shot noise.

Additionally, we performed SNR analysis (calculated by dividing the signal intensity by the standard deviation of the background) on synaptic puncta as we did previously for expansion revealing (ExR). In particular, we analyzed SNR of synapses that were identified based on joint RIM1/2 and PSD95 presence, in post-expansion antibody stained 20ExM brain tissue (same images as in Fig. 3b) and found that the SNR was ~35 (Supp. Fig. 2d). Although we were not able to use the same primary and secondary antibodies that were used for the post-expansion antibody staining ExR paper (the RIM1/2 primary antibody used in the ExR paper was discontinued), the SNR of synapses that were identified through staining of Bassoon, Cav2.1,

Homer1, PSD95, RIM1/2, Shank3, and SynGAP with the ExR protocol was on average ~15 (just to get a ballpark estimate, we averaged the SNR across all antibodies, using previously published data; source data: Extended Fig. 2d from the ExR paper, *Nat. Biomed. Eng.* **2022**, *6* (9), 1057–1073). Thus we estimate that our SNR is comparable to those of earlier technologies, with our current goal simply being to make the process easier, by enabling it to occur in a single step, rather than requiring repeated steps.

20-fold enlargement of a stained sample will dilute signal intensity greatly. Indeed, after 20-fold expansion, we expect an 8000-fold increase in volume, and corresponding 8000x decrease in fluorophore concentration. As the pre-expansion raw signal intensity of cell culture microtubule staining ranged from 15000 to 30000 (Supp. Fig. 2a), we would expect the intensity to drop to ~2–4 after 8000-fold volumetric dilution. Indeed, after expansion, we could not observe any remaining fluorescence without amplification.

With amplification, after tertiary antibody staining, we observed post-expansion signal intensity to be above 200, a ~100-fold increase in signal intensity compared to the expected diluted intensity (Supp. Fig. 2b; note that Supp. Fig. 2a and 2b were acquired under identical microscope settings and processed identically). The signal intensity was sufficient to reveal clear hollow microtubule structures, and support distortion and resolution analyses. We used post-expansion antibody staining to achieve sufficient SNR for our purposes. We note that SNR could, in principle, be further improved with any one of a number of previously published signal amplification methods, such as hybridization chain reaction (HCR) and rolling circle amplification (RCA), which, as modular DNA-based methods, have easily been incorporated into ExM protocols by multiple groups.

The apparent difference in noise between the low-magnification and zoomed-in images in Fig 3b was due to the z-projections of these images being conducted over different depths. Specifically, the low-magnification image was z-projected across the entire z range of the slice, whereas the zoomed-in images only contained ranges that contained a particular synapse. We have demonstrated this difference by showing the same synapses z-projected over the whole imaging range vs. the synapse-limited range, in Supp. Fig. 2f.

In the nanocolumn analysis, synapses were chosen based on the juxtaposition of RIM1/2 and PSD95 signals, as previously utilized in the ExR study. Signal-to-noise ratio (SNR) measurements, conducted as in the ExR study, revealed an SNR for 20ExM comparable to that of ExR, with both studies focused on synaptic proteins that are known to participate in nanocolumns. For Fig. 3b, we increased contrast to highlight the boundary of the synapses, not uncommon for studies emphasizing synaptic protein density shape, so we could easily identify synapses for subsequent data analysis. This does, notably, lead to many pixels within the synapses appearing saturated. We now also include the same images but with contrast adjusted to only have 1 pixel saturated per channel per image in Supp. Fig. 2e, highlighting the internal heterogeneity within the signal distribution of RIM1/2 and PSD95. While the synapse protein

gap, and the synaptic protein density shapes, were qualitatively similar, different contrast adjustments will of course emphasize different aspects of the data.

Supplementary Note 7: Expansion Factor and Resolution

20ExM revealed the hollow structure of microtubules. According to previous work, visualizing the hollow structure of microtubules requires at least 15x expansion, equivalent to 16–26 nm effective resolution (Fig. S2 from X10, *EMBO Rep.* **2018**, *19* (9). https://doi.org/10.15252/embr.201845836).

We have also added nuclear pore complex (NPC) images (see Ext Data Fig 5) where 20ExM resolved individual corners of NPCs which are around 42 nm apart from each other based on previous cryo-EM data. See Supp. Fig. 5 for visual comparisons of the current work with previous work on 20x expansion of NPCs.

Furthermore, 20ExM images of RIM1/2 and PSD95 also revealed that synaptic nanocolumns align with each other with 20–25-nm precision (Fig. 3e,f), also approaching the measured resolution, and matching the precision characterized by expansion revealing (see Fig. 4i and 4k of that earlier paper). Finally, the estimated nanoscale error introduced by expansion was about 10 nm, consistent with that of iExM, suggesting that for things larger than this size, the expansion factor - in this case, ~20x - should determine the resolution (300 nm / 20 ~ 15 nm).

Supplementary Note 8: Z-axis Isotropy

We imaged pre-expanded tissue with a 40x lens and expanded tissue with a 4x lens to ensure similar fields of view for downstream registration. While we made our best attempts to ensure samples were in the same orientation during confocal imaging before and after expansion, slight differences (less than 10˚ rotational difference, based on our visual examination) occurred. While miniscule, the slight rotation of the sample during confocal imaging before vs. after expansion resulted in a slight angle difference in the xz- and yz- plane that we selected in the sample's pre- and post-expansion images. We found this challenging to correct computationally. Indeed, in almost all ExM papers that compare pre- and post-expansion images, there is some difference between the appearance of the two images, due to this aspect of the imaging process: it is a reality of imaging the same sample twice. Despite the slight rotation, the overall shapes of soma and dendrites are similar between pre- and post-20ExM images.

Despite these challenges, we were able to perform distortion analysis across z-depths, comparing pre- and post-expanded tissue. We observed ~5% distortion over a distance of 15 μm, consistent with the distortion observed in the xy-plane (Supp. Fig. 3).

Supplementary Note 9: Quantitative Distance Measurements

To calculate expansion factor: many groups perform a pre-ExM low-magnification check of overall sample size and/or the dimensions of key features, for comparison to post-expansion measurements of the same features, and thus expansion factor calculation. To calculate expansion factor, please note that registration is not needed. Instead, users can simply measure the distance from one boundary of the sample to another, or from one biological landmark to another, and then compare that measurement between pre- and post-expansion states, as previously reported for earlier ExM methods (*Science* **2015**, *347* (6221), 543–548, *Nat. Biotechnol.* **2016**, *34* (9), 987–992). This kind of measurement is necessary for quantitative measurement, and we have provided instructions in our 2018 protocols paper (*Curr. Protoc. Cell Biol.* **2018**, *80* (1), e56).
