# Supplementary material for: Single-shot 20-fold expansion microscopy
Source: Nat Methods. 2024 Oct 11;21(11):2128–34. doi: 10.1038/s41592-024-02454-9 (PMC11541206; doi:10.1038/s41592-024-02454-9)
Supplement: Supplementary file 2 — Reporting Summary [file 41592_2024_2454_MOESM2_ESM.pdf]

Corresponding author(s): Edward Boyden, Laura Kiessling

Last updated by author(s): 7/26/2024

Reporting Summary

Nature Portfolio wishes to improve the reproducibility of the work that we publish. This form provides structure for consistency and transparency in reporting. For further information on Nature Portfolio policies, see our Editorial Policies and the Editorial Policy Checklist.

Statistics

For all statistical analyses, confirm that the following items are present in the figure legend, table legend, main text, or Methods section.

n/a	Confirmed	
☐	☒	The exact sample size (n) for each experimental group/condition, given as a discrete number and unit of measurement
☐	☒	A statement on whether measurements were taken from distinct samples or whether the same sample was measured repeatedly
☒	☐	The statistical test(s) used AND whether they are one- or two-sided<br>*Only common tests should be described solely by name; describe more complex techniques in the Methods section.*
☒	☐	A description of all covariates tested
☒	☐	A description of any assumptions or corrections, such as tests of normality and adjustment for multiple comparisons
☐	☒	A full description of the statistical parameters including central tendency (e.g. means) or other basic estimates (e.g. regression coefficient) AND variation (e.g. standard deviation) or associated estimates of uncertainty (e.g. confidence intervals)
☒	☐	For null hypothesis testing, the test statistic (e.g. F, t, r) with confidence intervals, effect sizes, degrees of freedom and P value noted<br>*Give P values as exact values whenever suitable.*
☒	☐	For Bayesian analysis, information on the choice of priors and Markov chain Monte Carlo settings
☒	☐	For hierarchical and complex designs, identification of the appropriate level for tests and full reporting of outcomes
☒	☐	Estimates of effect sizes (e.g. Cohen's d, Pearson's r), indicating how they were calculated

Our web collection on statistics for biologists contains articles on many of the points above.

Software and code

Policy information about availability of computer code

Data collection	Spinning disk confocal microscope with NIS-Element Advanced Research
Data analysis	Python (version 3.11.7), Fiji (version 2.14.0/1.54f; Built-in: Rolling Ball Function, Gaussian Filter Function, Auto-scaling Function, Line Selection Tool; Plug-in: Radial Profile 1.0, NanoJ-SQUIRREL 1.0), Microsoft Excel (version 16.87), MATLAB (version R2020a). Custom codes used for Distortion Analysis, Peak-to-peak Distance Analysis, and Autocorrelation and Enrichment Analysis are available on GitHub at https://github.com/shiwei-w/20ExM.

For manuscripts utilizing custom algorithms or software that are central to the research but not yet described in published literature, software must be made available to editors and reviewers. We strongly encourage code deposition in a community repository (e.g. GitHub). See the Nature Portfolio guidelines for submitting code & software for further information.

Data

Policy information about availability of data

All manuscripts must include a data availability statement. This statement should provide the following information, where applicable:

- Accession codes, unique identifiers, or web links for publicly available datasets
- A description of any restrictions on data availability
- For clinical datasets or third party data, please ensure that the statement adheres to our policy

Source and processed imaging data generated in this study is available on Open Science Framework at https://osf.io/kezgs. The source data used for synaptic

nanocolumn analysis is available on GitHub at https://github.com/shiwei-w/20ExM.

Human research participants

Policy information about studies involving human research participants and Sex and Gender in Research.

Reporting on sex and gender	N/A
Population characteristics	N/A
Recruitment	N/A
Ethics oversight	N/A

Note that full information on the approval of the study protocol must also be provided in the manuscript.

Field-specific reporting

Please select the one below that is the best fit for your research. If you are not sure, read the appropriate sections before making your selection.

☒ Life sciences ☐ Behavioural & social sciences ☐ Ecological, evolutionary & environmental sciences

For a reference copy of the document with all sections, see nature.com/documents/nr-reporting-summary-flat.pdf

Life sciences study design

All studies must disclose on these points even when the disclosure is negative.

Sample size	We have clearly described the number of samples we tried for each experiments and results. All of the conclusions were based on n>1 replications. As we are demonstrating a new technology, we performed two to three biological replicates with the same protocol to demonstrate the reproducibility of the protocol.
Data exclusions	None
Replication	For all experiments, we performed two to three independent biological replicates. All the replications were successful.
Randomization	Since we are just demonstrating our technology, instead of, for example, determining the differences of treatment and control groups, randomization was not relevant to this study.
Blinding	Since we are just demonstrating our technology, instead of, for example, determining the differences of treatment and control groups, blinding was not relevant to this study.

Reporting for specific materials, systems and methods

We require information from authors about some types of materials, experimental systems and methods used in many studies. Here, indicate whether each material, system or method listed is relevant to your study. If you are not sure if a list item applies to your research, read the appropriate section before selecting a response.

Materials & experimental systems

n/a	Involved in the study	
☐	☒	Antibodies
☐	☒	Eukaryotic cell lines
☒	☐	Palaeontology and archaeology
☐	☒	Animals and other organisms
☒	☐	Clinical data
☒	☐	Dual use research of concern

Methods

n/a	Involved in the study	
☒	☐	ChIP-seq
☒	☐	Flow cytometry
☒	☐	MRI-based neuroimaging

Antibodies

Antibodies used	anti-Beta tubulin (Rabbit, Abcam, ab6046),

Antibodies used	anti-mNeonGreen (Mouse, Proteintech, 32f6),<br>anti-TOM20 (Rabbit, Proteintech, 11802-1-AP),<br>anti-rabbit Alexa Fluor 546 (Goat, ThermoFisher, A11035)<br>anti-mouse Alexa Fluor 568 (Goat, ThermoFisher, A21043)<br>anti-goat Alexa Fluor Plus 555 (Donkey, ThermoFisher, A32816)<br>anti-RIM1/2 (Guinea pig, Synaptic Systems, 140205)<br>anti-PSD95 (Mouse, ThermoFisher, MA1-046)<br>anti-GFP (Rabbit, ThermoFisher, A11122)<br>anti-Guinea pig Alexa Fluor 555 (Goat, ThermoFisher, A21435)<br>anti-Mouse Alexa Fluor Plus 647 (Donkey, ThermoFisher, A32787)<br>anti-Rabbit Alexa Fluor Plus 488 (Goat, ThermoFisher, A11008)
Validation	All of the antibodies are commercially available. The following validations are performed by the vendors.<br>anti-Beta tubulin (Rabbit, Abcam, ab6046): Validated for Human IHC-P, ICC/IF, IP, and WB<br>anti-mNeonGreen (Mouse, Proteintech, 32f6): Validated for Mouse ICC/IF and ELISA<br>anti-TOM20 (Rabbit, Proteintech, 11802-1-AP): Validated for Human WB, IP, and ICC/IF, and Mouse WB, IHC<br>anti-RIM1/2 (Guinea pig, Synaptic Systems, 140205): Validated for Rat WB and ICC/IF, and Mouse ExM IHC<br>anti-PSD95 (Mouse, ThermoFisher, MA1-046): Validated for Mouse WB, Rat WB and ICC/IF, Human ICC/IF<br>anti-GFP (Rabbit, ThermoFisher, A11122): Validated for Human WB and ICC/IF

Eukaryotic cell lines

Policy information about cell lines and Sex and Gender in Research

Cell line source(s)	HEK 293 from ThermoFisher, catalog no. R70007; Human colorectal adenocarcinoma cells (DLD-1) with homozygous insertion at Nup96 loci containing NeonGreen moiety and an auxin-inducible degron (Nup96::Neon-AID) from laboratory of T. Schwartz and M. Dasso (Ref. 21: Regmi et al., 2020; Ref. 22: Schuller et al., 2021).
Authentication	HEK 293 are available at ThermoFisher, catalog no. R70007, Authentication method not specified by vendor;<br>Creation and authentication of the Nup96::Neon-AID DLD-1 cell line was performed in a separate study (Ref. 21: Regmi et al., 2020), Authenticated by PCR assays with insertion-specific primers, imaging assay, and Auxin activation assay.
Mycoplasma contamination	The cell lines were not tested for mycoplasma contamination.
Commonly misidentified lines<br>(See ICLAC register)	No commonly misidentified lines were used in the study.

Animals and other research organisms

Policy information about studies involving animals; ARRIVE guidelines recommended for reporting animal research, and Sex and Gender in Research

Laboratory animals	Thy1-YFP-H, 6–8 weeks of age.
Wild animals	No wild animals were used in the study.
Reporting on sex	Used without regard to sex.
Field-collected samples	No field-collected samples were used in the study.
Ethics oversight	All procedures involving mice (Thy1-YFP-H, 6–8 weeks of age from JAX, used without regard to sex) were performed in accordance with the US National Institutes of Health Guide for the Care and Use of Laboratory Animals and approved by the MIT Committee on Animal Care.

Note that full information on the approval of the study protocol must also be provided in the manuscript.
